# Comparison of Different Recovery Strategies After High-Intensity Functional Training: A Crossover Randomized Controlled Trial

**DOI:** 10.3389/fphys.2022.819588

**Published:** 2022-02-03

**Authors:** Rafael Martínez-Gómez, Pedro L. Valenzuela, Alejandro Lucia, David Barranco-Gil

**Affiliations:** ^1^Faculty of Sports Sciences, Universidad Europea de Madrid, Madrid, Spain; ^2^Department of Sport and Health, Spanish Agency for Health Protection in Sport (AEPSAD), Madrid, Spain; ^3^Physical Activity and Health Research Group (PaHerg), Instituto de Investigación Sanitaria Hospital ‘12 de Octubre’ (‘imas12’), Madrid, Spain

**Keywords:** performance, fatigue, CrossFit, exercise, electrical stimulation

## Abstract

We aimed to determine whether voluntary exercise or surface neuromuscular electrical stimulation (NMES) could enhance recovery after a high-intensity functional training (HIFT) session compared with total rest. The study followed a crossover design. Fifteen male recreational CrossFit athletes (29 ± 8 years) performed a HIFT session and were randomized to recover for 15 min with either low-intensity leg pedaling (“Exercise”), NMES to the lower limbs (“NMES”), or total rest (“Control”). Perceptual [rating of perceived exertion (RPE) and delayed-onset muscle soreness (DOMS) of the lower-limb muscles], physiological (heart rate, blood lactate and muscle oxygen saturation) and performance (jump ability) indicators of recovery were assessed at baseline and at different time points during recovery up to 24 h post-exercise. A significant interaction effect was found for RPE (*p* = 0.035), and although *post hoc* analyses revealed no significant differences across conditions, there was a quasi-significant (*p* = 0.061) trend toward a lower RPE with NMES compared with Control immediately after the 15-min recovery. No significant interaction effect was found for the remainder of outcomes (all *p* > 0.05). Except for a trend toward an improved perceived recovery with NMES compared with Control, low-intensity exercise, NMES, and total rest seem to promote a comparable recovery after a HIFT session.

## Introduction

Enhancing recovery between workouts is a key issue in competition sports, as it might allow athletes to cope (and adapt to) increasing training loads, ultimately contributing to an improved performance (Bishop et al., 2008). A fast recovery is even more important in those sports where athletes must face consecutive competition days or even different competition sessions in the same day (Bishop et al., 2008). Therefore, identifying methods that could foster recovery between sessions is of major relevance (Maté-Muñoz et al., 2017).

High-intensity functional training (HIFT, i.e., training programs that incorporate functional and multimodal movements performed at relatively high intensities) has become a popular exercise modality in recent years (Feito et al., 2018), with CrossFit among the most popular examples (Claudino et al., 2018). Different studies have shown that HIFT sessions induce remarkable levels of fatigue, as reflected by an impairment of performance indicators (e.g., 1 repetition maximum, jump height, rate of force development), increased levels of biomarkers such as blood lactate or creatine kinase, and high values of perceptual fatigue (Maté-Muñoz et al., 2017; Timón et al., 2019). Indeed, a greater fatigue has been reported to occur after HIFT sessions compared with more “traditional” training sessions (Drum et al., 2017). However, despite the popularity of HIFT and its highly fatiguing nature, scarce evidence exists on which strategies could enhance recovery after this training modality.

A wide variety of strategies are commonly used by athletes of different sports to optimize recovery between exercise sessions (Reilly and Ekblom, 2005; Barnett, 2006; Bishop et al., 2008). Strong evidence suggests that active recovery, mainly low-intensity exercise, might be more effective than total rest (Signorile et al., 1993; Connolly et al., 2003). However, in practical terms performing actual exercise between sessions or competitions is not always feasible. In this effect, passive strategies such as low-frequency surface neuromuscular electrical stimulation (NMES, which elicits low-intensity involuntary muscle contractions through the application of intermittent electrical stimuli to skeletal muscles) might be a potentially effective recovery strategy, at least in part due to an improved blood flow and metabolite removal (Babault et al., 2011). Controversy exists, however, on the effectiveness of NMES as a recovery strategy, and indeed a meta-analysis reported mixed or no evidence compared to either passive or active recovery (Malone et al., 2014a). Later studies have reported a beneficial effect of post-exercise NMES over passive recovery on different outcomes including muscle inflow, lactate removal, or performance (Bieuzen et al., 2014; Taylor et al., 2015; Borne et al., 2017), although other authors have found similar effects with both strategies (Malone et al., 2012, 2014b). For instance, Malone et al. compared the effects of 30 min of NMES, active recovery (low-intensity cycling) and passive recovery after high-intensity intermittent exercise (consecutive Wingate anaerobic tests) in healthy trained male triathletes, and found a higher blood lactate removal with active recovery but overall comparable effects on performance across all recovery modalities (Malone et al., 2012). Another study reported no differences in blood lactate removal, perceived muscle soreness or performance between active recovery (walking), NMES or massage in healthy amateur athletes after a single bout of high intensity training (Akinci et al., 2020). Thus, evidence on whether NMES could provide superior benefits to total rest or comparable benefits to those induced by active recovery is mixed and scarce (Malone et al., 2012; Paradis-Deschênes et al., 2020).

The aim of the present study was to compare the effects of three different recovery strategies [active recovery (voluntary exercise), NMES, or total rest] following a HIFT session. Our main outcome was performance (i.e., jump height), but we also aimed to measure other secondary outcomes including subjective (e.g., perceived exertion) and physiological (e.g., blood lactate) measures of recovery. Following previous research (Malone et al., 2014a), we hypothesized that both NMES and active recovery would induce similar benefits on perceptual measures of recovery—in both cases superior to total rest—while no differences would be observed between conditions on performance measures.

## Materials and Methods

### Subjects

Fifteen recreational male athletes from a local CrossFit center volunteered to participate [age (mean ± SD): 29 ± 8 years, weight: 81 ± 12 kg, height: 177 ± 6 cm]. All participants had previous training experience with HIFT (≥1 year, ≥3 training sessions/week) and were familiarized with all the exercises and testing procedures of our protocol. During the study, participants maintained their regular training program and dietary pattern, but were required to refrain from exercising or consuming ergogenic aids/stimulants (e.g., creatine, caffeine) ≥24 h and ≥72 h before and after each testing session, respectively. Participants provided written informed consent, and all procedures were conducted following the standards set by the Declaration of Helsinki and its later amendments. The study was approved by the Institutional Review Board (*Hospital Universitario Fundación Alcorcón*, Spain; #19/51).

### Experimental Design

**FIGURE 1:**
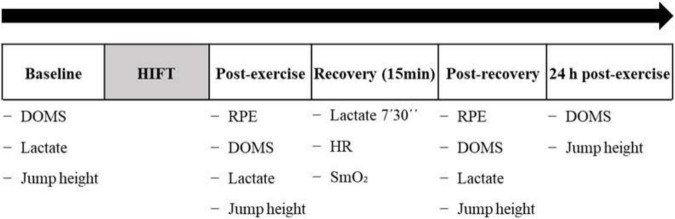
Schematic figure representing the experimental protocol. Abbreviations: DOMS, delayed-onset muscle soreness; HR, heart rate; RPE, rating of perceived exertion; SmO_2_, muscle oxygen saturation.

The present study followed a crossover randomized controlled trial design. A summary of the experimental protocol is shown in Figure 1. Participants performed a HIFT session on three occasions, each session from the next one by a minimum of 72 h and a maximum of one week. Participants were randomized using computer-generated random numbers to recover for 15 min after each HIFT session with either voluntary exercise (*Exercise*, low-intensity leg pedaling), passive muscle contractions (NMES to the lower limbs), or a control condition (*Control*, total rest).

### Training Sessions

All training sessions were supervised by a specialist coach, who provided standardized encouragement and was blinded to participants’ recovery conditions. Before each individual session participants performed a warm-up consisting of 5 min of low-intensity leg pedaling [rating of perceived exertion (RPE) of 6 out of 10] (Borg, 1982), 5 min of joint mobility and stability exercises, and 5 min of specific exercises (five push presses, five front squats, and five thrusters, respectively, first with a 20-kg bar and thereafter with a 43-kg bar). The main part of the HIFT session consisted of the Fran workout, a benchmark workout of the day (WOD) within CrossFit. This specific WOD consists of two exercises (thrusters with a loaded barbell of 43 kg and pull-ups) performed in alternating fashion in a descending 21-15-9 repetition scheme. That is, individuals completed 21 repetitions of thrusters followed by 21 repetitions of pull-ups, then 15 repetitions of these two exercises, and finally nine repetitions. The time needed to complete the WOD was registered.

### Recovery Methods

Following the WOD, participants recovered for 15 min with one of the three aforementioned strategies. During the control condition, participants remained seated for 15 min. During exercise, participants performed low-intensity leg pedaling (RPE of 6 out 10) on a cycle ergometer (Assault Fitness, Rogue Fitness Europe, Pori, Finland) with a self-selected cadence (Menzies et al., 2010). During NMES, an electrical stimulator (Compex SP 8.0, Geneva, Switzerland) with surface electrodes (5 × 5 cm, axion^®^ GmBh, Leonberg, Germany) was used to evoke involuntary muscle contractions. We used six channels (three per leg) and two electrodes per channel, which were placed on the origin and muscle belly of the quadriceps (∼2/3 of the rectus femoris), hamstrings (∼2/3 of the biceps femoris and semitendinosus), and calves (∼2/3 of both gastrocnemius to Achilles’ tendon) of both legs. We used a current of 5 Hz with a pulse duration of 300 μs at an individualized intensity so as to evoke visible muscle contractions without generating pain (i.e., intensity was increased until the device indicated that it had reached the minimum intensity that produced therapeutic effects), resulting in an average intensity of 22 ± 9 (range 9–41) and 23 ± 10 (range 9–42) mA for the left and right leg, respectively (Malone et al., 2014a).

### Measures

#### Subjective Measures

Exercise-induced delayed-onset of muscle soreness (DOMS) of the lower-limb muscles was assessed at four time points (baseline, immediately post-WOD, post-recovery, and 24 h post-exercise, respectively) through a 0–10 visual analog scale (VAS) while participants performed a squat holding a 90-degree knee position for 5 s (Barnett, 2006). RPE was assessed on a 0–10 scale (Borg, 1982) post-WOD and post-recovery.

#### Physiological Measures

Blood lactate concentration was quantified using a portable analyzer (Lactate Scout, SensLab GmbH; Leipzig, Germany). Fingertip capillary blood samples (0.5 μL) were taken at baseline (before warm-up) and at several time points during the recovery phase (0, 7.5, and 15 min, respectively, after the WOD). Muscle oxygen saturation (SmO_2_) of the right *vastus lateralis* muscle was determined continuously during the 15 min of the recovery phase (Humon, Cambridge, MA, United States) (Farzam et al., 2018). Heart rate (HR) was continuously monitored during the recovery phase with a chest band (BerryKing, BK-HB16-01; Herne, Germany) connected to a mobile app (Wahoo for iPhone 7, Apple Inc., CA, United States).

#### Performance Measures

Jump height attained in a countermovement (CMJ) and drop jump (DJ) was measured at baseline, post-WOD, immediately post-recovery and 24 h post-exercise, respectively, using a validated mobile app (MyJump2 for iPhone 7, Apple Inc., CA, United States) (Balsalobre-Fernández et al., 2014; Haynes et al., 2019). Participants performed three trials for both CMJ and DJ, with the best results used for analysis. During the CMJ, participants performed a downward movement and jumped when reaching a knee angle of ∼90°. For the DJ, participants stepped from a 40-cm bench and jumped as high as possible with the minimal possible ground contact time. Reactive strength index (RSI) was calculated as jump height in the DJ divided by contact time. Participants were instructed not to flex their knees during flight or landing phases (to avoid an overestimation of flight time) and to maintain their hands on their hips while performing the jumps.

### Statistical Analysis

Based on the effect size [partial eta squared (η_p_^2^) = 0.217] reported by a previous study for the effect of NMES applied after a high-intensity training session on jump performance (Taylor et al., 2015), using GPower (version 3.1.9.2, Universität Düsseldorf, Germany) we estimated that a sample size of 15 participants would be appropriate to find significant differences between conditions in a within-subject research design (β > 80%, α < 0.05, number of groups = 3, number of measurements = 4).

Data are shown as mean ± SD. The normality and homoscedasticity of the data was tested using the Kolmogorov-Smirnov and Levene’s test, respectively. Differences between recovery strategies were determined with a two-way repeated measures ANOVA, with both condition (i.e., recovery strategy) and time as within-subject factors. In order to minimize the risk of type I error, *post hoc* analyses (Bonferroni test) were performed only when a significant interaction (time by recovery strategy) effect was found. Effect sizes (η_p_^2^) were also computed. All analyses were performed using SPSS (version 23.0, Armonk, NY, United States) setting the level of significance at *p* < 0.05.

## Results

All participants completed the WOD. No significant differences were found between recovery methods for the time needed to complete the WOD (340 ± 101, 338 ± 101 and 315 ± 66 s for control, exercise and NMES, respectively; *p* = 0.410; η_p_^2^ = 0.062), and RPE reported immediately after the WOD (8.7 ± 0.9, 9.2 ± 1.0 and 9.0 ± 0.8 arbitrary units, respectively; *p* = 0.106; η_p_^2^ = 0.148), suggesting similar intensity levels for the three conditions.

When analyzed regardless of the experimental condition applied, no differences were found between sessions for performance nor for any analyzed outcome (all *p* > 0.3, data not shown), which suggests that there was no accumulated fatigue nor a learning/familiarization effect.

### Subjective Measures

**FIGURE 2:**
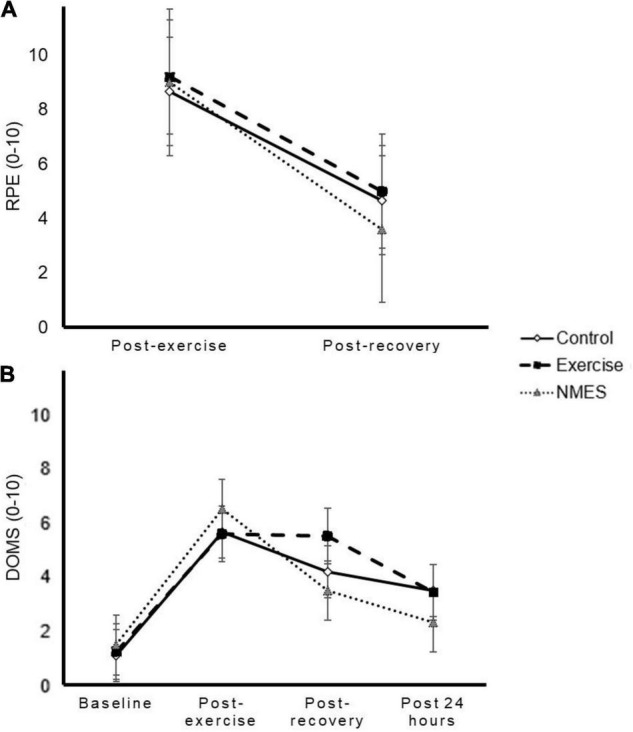
Effects of low-intensity exercise (Exercise), surface neuromuscular electrical stimulation (NMES), or total rest (Control) on rating of perceived exertion (RPE, panel **A**) and delayed-onset muscle soreness (DOMS, panel **B**). A significant interaction effect was found for both variables (*p* = 0.035 and *p* = 0.017, respectively), but *post hoc analyses* revealed no significant differences at any time point.

All three strategies resulted in a reduced RPE at post-recovery compared with post-exercise (time effect *p* < 0.001). In turn, a significant time by strategy interaction effect was observed (*p* = 0.035; η_p_^2^ = 0.213) with a non-significant trend toward a lower RPE with NMES compared with control (*p* = 0.061) but with no differences between NMES and exercise or between control and exercise (Figure 2A). DOMS increased above baseline values at post-exercise, post-recovery and 24 h later, respectively (time effect *p* < 0.001) and a significant interaction effect was observed (*p* = 0.017; η_p_^2^ = 0.164, Figure 2B). However, *post hoc* analyses revealed not pairwise differences across conditions at any time point (*p* > 0.05).

### Physiological Measures

**FIGURE 3:**
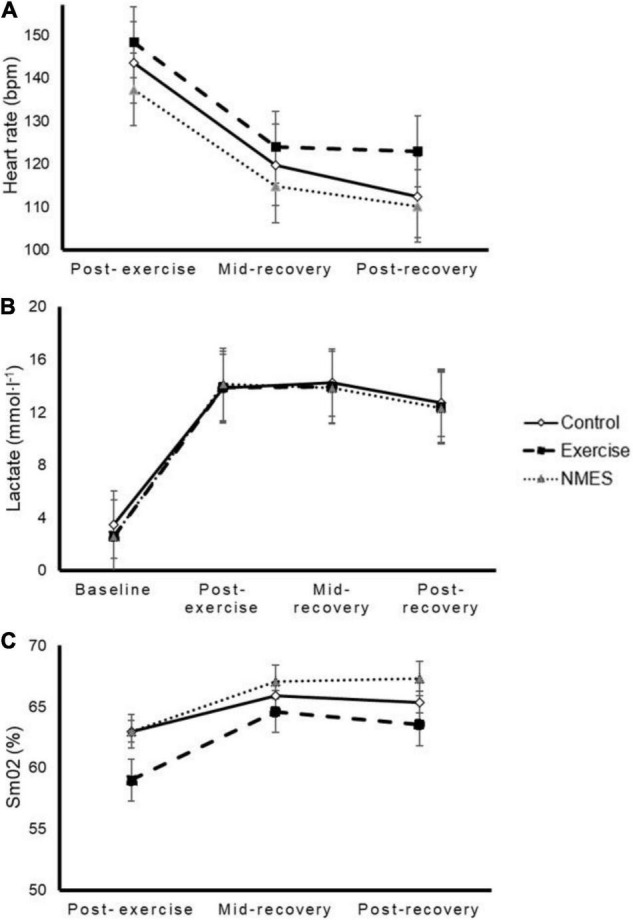
Effects of low-intensity exercise (Exercise), surface neuromuscular electrical stimulation (NMES) or total rest (Control) on heart rate (panel **A**), blood lactate (panel **B**), and muscle oxygen saturation (SmO_2_, panel **C**). No significant interaction effect was found (*p* = 0.920, *p* = 0.831, and *p* = 0.694, respectively).

A significant time (*p* < 0.001, *p* = 0.004, and *p* = 0.030, respectively) but no significant interaction effect [*p* = 0.920 (η_p_^2^ = 0.023), *p* = 0.831 (η_p_^2^ = 0.026), and *p* = 0.694 (η_p_^2^ = 0.038)] was noted for blood lactate (Figure 3A), HR (Figure 3B), and SmO_2_ (Figure 3C).

### Performance Measures

**FIGURE 4:**
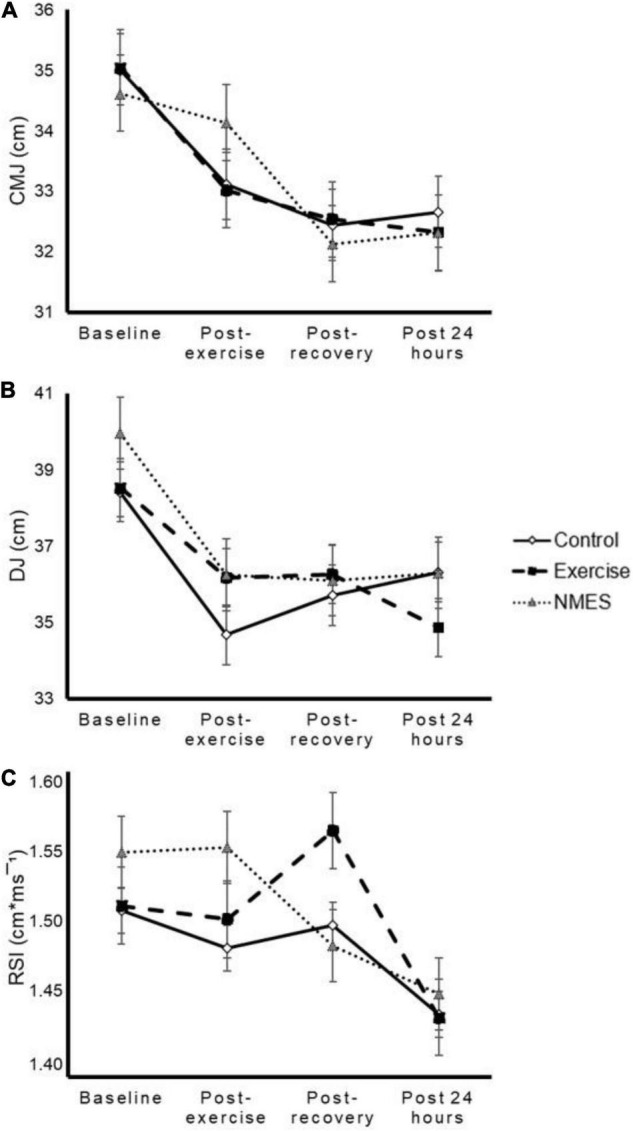
Effects of low-intensity exercise (Exercise), neuromuscular electrical stimulation (NMES), or total rest (Control) on countermovement jump (CMJ, panel **A**), drop jump (DJ, panel **B**), and reactive strength index (RSI, panel **C**). No significant time by condition interaction effect was found (*p* = 0.388, *p* = 0.296, and *p* = 0.390, respectively).

Jump performance significantly declined after exercise and kept below baseline levels after the recovery phase and 24 h later [time effect *p* < 0.001, *p* < 0.001, and *p* = 0.059 for CMJ (Figure 4A), DJ (Figure 4B), and RSI (Figure 4C)]. However, no differences were found between methods [interaction effect *p* = 0.388 (η_p_^2^ = 0.071), *p* = 0.296 (η_p_^2^ = 0.081), and *p* = 0.390 (η_p_^2^ = 0.071) for CMJ (Figure 4A), DJ (Figure 4B), and RSI (Figure 4C), respectively].

## Discussion

Our findings suggest a comparable effectiveness of NMES, low-intensity exercise or total rest for enhancing recovery after HIFT, with the former tending to lower perceived fatigue immediately after recovery compared with total rest. However, no additional benefits were found with NMES for other perceptual indicators (DOMS) or for physiological (blood lactate, HR, muscle oxygen kinetics) or performance (jump performance) outcomes.

Previous studies comparing the effectiveness of NMES and active recovery have yielded conflicting results (Heyman et al., 2009; Malone et al., 2012; Bieuzen et al., 2014; Taylor et al., 2015). Neric et al. (2009) found that, when applied with low-frequency, NMES might accelerate the removal of metabolites such as lactate compared with active recovery (sub-maximal swimming) after a sprint swim (200 yard). However, several studies have found no benefits with NMES or even lower benefits than those elicited by active recovery. Akinci et al. (2020) reported no differences between NMES and active recovery (walking at 40% heart rate reserve) on muscle strength, DOMS, or blood lactate removal after a HIT session. More recently, Paradis-Deschênes et al. (2020) reported no differences on muscle oxygen kinetics, blood lactate concentration, pH or performance when recovering with NMES or low-intensity exercise (cycling) between two consecutive 5-km cycling time trials. Other studies have also reported no beneficial effects on performance nor on other fatigue indicators such as heart rate, RPE, blood lactate, or DOMS with NMES or active recovery after a futsal game or a high-intensity exercise bout (Lattier et al., 2004; Tessitore et al., 2008). Moreover, Bieuzen et al. reported a better short-term recovery between two bouts of exhausting exercise (Yo-Yo Intermittent Recovery tests) in female handball players with active recovery (low-intensity cycling) compared with NMES (Bieuzen et al., 2014). In the same line, Malone et al. reported an impaired blood lactate clearance and no benefits (or even performance impairments) with NMES recovery (30 min of self-intensity) compared with active recovery [30 min cycling at 30% maximal oxygen consumption (VO_2max_)] between consecutive high-intensity exercise bouts (consecutive Wingate Anaerobic tests) (Malone et al., 2012, 2014a).

Some controversy also exists regarding a hypothetical superiority of NMES over total rest. Borne et al. reported that, among several recovery strategies (total rest, blood flow restriction, placebo, NMES) applied after consecutive 30-s bouts of supramaximal exercise, NMES elicited the largest increases in calf arterial inflow and was the only one that allowed recovery of performance between exercise bouts (Borne et al., 2017). Other studies have provided further support to these findings. Notably, Bieuzen et al. showed that, after a high-intensity repeated-sprint test, NMES applied to the calf muscles for 15 min accelerated the return of pH and blood lactate to baseline values compared with total rest, also improving performance recovery (Bieuzen et al., 2014). Taylor et al. (2015) reported that NMES fostered performance recovery and resulted in lower levels of serum creatine kinase (an indicator of skeletal muscle damage) and muscle soreness 24 h after a repeated-sprints training session compared with total rest. Also, Barcala-Furelos et al. (2020) concluded that NMES applied immediately after a water rescue (200 m swimming with “false human victims”) could be an effective recovery strategy to clear out blood lactate compared with total rest. In the present study, we observed a quasi-significant trend (*p* = 0.061) toward a greater perceived recovery with NMES compared with total rest, which is in line with previous research (Tessitore et al., 2008; Cortis et al., 2010). However, no benefits were found for any of the remaining outcomes. Similarly, Malone et al. (2012, 2014b) reported no differences when using NMES or total rest as recovery, on performance or different physiological markers (blood lactate, HR). Thus, further evidence is needed to support an eventual superiority of NMES over total rest for recovery after strenuous exercise, although the potential benefits we found on perceived recovery—which could also be due, at least partly, to a certain placebo effect—should not be overlooked.

Interestingly, our findings also suggest no benefits of active recovery over total rest. One of the main reasons for supporting a potential benefit of active recovery versus rest is the increased blood flow with the former, which could accelerate metabolite removal (e.g., lactate) and increase oxygen and nutrient supply to the muscle. The recruitment of these muscle fibers was confirmed by the high blood lactate concentrations recorded in “Fran session” (14.0 mmol l^–1^) as Fernandez-Fernandez et al. reported about lactate responses to a CrossFit WOD with similar sample (Fernández et al., 2015). However, the practical relevance of a hastening blood lactate removal in terms of recovery remains questionable (Van Hooren and Peake, 2018). Although controversy exists and some benefits have been reported particularly on subjective measures (perceived recovery) (Ortiz et al., 2019) at present there is no consistent evidence supporting the superiority of active post-exercise recovery over total rest on physiological or performance parameters (Van Hooren and Peake, 2018).

Several factors could at least partly explain the lack of beneficial effects observed in the present study with NMES or even with active recovery compared with total rest. We observed no benefits after an exercise bout (“Fran WOD”) that is physically demanding, as reflected by high RPE (∼9 out of 10) and lactate values (>12 mmol l^–1^)—which is in line with the responses reported by other authors for the same WOD (Fernández et al., 2015). However, whether these strategies could be beneficial after other types of WOD requires further investigation. In this regard, it is important to note that the WOD performed in the present study included both lower- and upper-limb exercises, whereas the recovery strategies applied did only target the lower limbs. Research is therefore warranted to confirm whether whole-body recovery strategies could provide greater benefits in this type of exercise. On the other hand, methodological factors such as the intensity or stimulation frequency of NMES, or the intensity and exercise modality of active recovery, could also potentially conditionate their effectiveness. Moreover, the short duration of the recovery phase (15 min) could also explain the lack of beneficial effects observed with both NMES and active recovery.

Some limitations of the present study should be noted, such as the fact that we did not assess some important fatigue-related variables (e.g., serum creatine kinase, muscle glycogen levels, upper-body DOMS) or sport-specific (i.e., HIFT) performance. Moreover, our findings are applicable to the present protocol and not necessarily generalizable to other exercise stimuli. In turn, a major strength is that, to our knowledge, this is the first study to assess the effectiveness of different recovery strategies after a HIFT session. The variety of outcomes we determined (including perceptual, physiological and performance indicators) can also be considered a strength.

## Conclusion

The present findings suggest that CrossFit athletes can attain a similar short-term recovery with either total rest, low-intensity exercise or NMES, with the former being in addition a simpler and more economical option. It must be noted, however, that NMES might result in a slightly, quasi-significant improvement in the subjective perception of recovery immediately after its application, although a potential placebo effect should not be disregarded.

## Data Availability Statement

The raw data supporting the conclusions of this article will be made available by the authors, without undue reservation.

## Ethics Statement

The studies involving human participants were reviewed and approved by Hospital Universitario Fundación Alcorcón. The patients/participants provided their written informed consent to participate in this study.

## Author Contributions

RM-G and PV contributed equally to realize the study. DB-G and AL contributed to the design, analysis of the results, and to the writing of the manuscript. All authors contributed to the article and approved the submitted version.

## Conflict of Interest

The authors declare that the research was conducted in the absence of any commercial or financial relationships that could be construed as a potential conflict of interest.

## Publisher’s Note

All claims expressed in this article are solely those of the authors and do not necessarily represent those of their affiliated organizations, or those of the publisher, the editors and the reviewers. Any product that may be evaluated in this article, or claim that may be made by its manufacturer, is not guaranteed or endorsed by the publisher.
